# A Novel Approach to Structure Plant-Based Yogurts Using High Pressure Processing

**DOI:** 10.3390/foods9081126

**Published:** 2020-08-15

**Authors:** Shaun Y. J. Sim, Xin Yi Hua, Christiani Jeyakumar Henry

**Affiliations:** 1 Clinical Nutrition Research Centre (CNRC), Singapore Institute of Food and Biotechnology Innovation (SIFBI), Agency for Science, Technology and Research (A*STAR), 14 Medical Drive, Singapore 117599, Singapore; Shaun_Sim@sifbi.a-star.edu.sg (S.Y.J.S.); Hua_Xin_Yi@sifbi.a-star.edu.sg (X.Y.H.); 2 Department of Biochemistry, Yong Loo Lin School of Medicine, National University of Singapore, 8 Medical Drive, Singapore 117596, Singapore

**Keywords:** high pressure processing, plant proteins, hydrogels, emulsion gels, plant-based yogurts

## Abstract

Current plant-based yogurts are made by the fermentation of plant-based milks. Although this imparts fermented flavors and probiotic cultures, the process is relatively longer and often leads to textural issues. The protein content of these plant-based yogurts is also lower than their dairy counterparts. To overcome these challenges, this paper explores the high pressure processing (HPP) of plant protein ingredients as an alternative structuring strategy for plant-based yogurts. Using mung bean (MB), chickpea (CP), pea (PP), lentil (LP), and faba bean (FB) proteins as examples, this work compared the viscosity and viscoelastic properties of high pressure-structured (600 MPa, 5 min, 5 °C) 12% (*w/w*) plant protein gels without, and with 5% (*w/w*) sunflower oil (SO) to commercial plain skim and whole milk Greek yogurts and discussed the feasibility of using HPP to develop plant-based yogurts. HPP formed viscoelastic gels (G’ > G’’) for all plant protein samples with comparable gel strength (G’~10^2^–10^3^ Pa; tan δ~0.2–0.3) to commercial dairy yogurts. The plant protein gel strength decreased in the order: CP~CPSO~LP~LPSO > MBSO~PPSO~FB~FBSO > PP >> MB. Modest addition of sunflower oil led to little change in viscoelastic properties for all plant protein samples except for MB and PP, where gel strength increased with incorporated oil. The emulsion gels were also more viscous than the hydrogels. Nonetheless, the viscosity of the plant protein gels was similar to the dairy yogurts. Finally, a process involving separate biotransformation for optimized flavor production and high pressure processing for consistent texture generation was proposed. This could lead to high protein plant-based yogurt products with desirable texture, flavor, and nutrition.

## 1. Introduction

The demand for plant-based alternatives to meat and dairy products has been increasing to alleviate the intense strain of animal husbandry on the environment [1]. Among the dairy substitutes, plant-based milks have received considerable attention and is currently widely available [2]. The texture of plant-based yogurts, however, could be improved to increase acceptability. At present, plant-based yogurt products adopt the traditional yogurt-making process through the fermentation of plant-based milks [3,4]. While this imparts fermented flavors and probiotic cultures, the acidification of plant proteins often leads to weak gel formation and phase separation [5]. Hydrocolloids are thus typically included to stabilize and enhance the texture, which is not desirable due to clean label reasons [6]. Besides having a longer (4–24 h) fermentation period, the protein content of these plant-based yogurts is also lower than their dairy counterparts. Therefore, traditional fermentation methods may not be optimal for plant-based yogurts. 

This presents opportunities to rethink the yogurt-making process to better suit plant-based systems. This paper explores the high pressure processing (HPP) of plant protein ingredients as an alternative structuring strategy for plant-based yogurts. HPP is a nonthermal processing method primarily used to extend the shelf-life of food products by subjecting foods to high hydrostatic pressure between 200–800 MPa [7]. HPP can also form homogenous plant protein gels with minimal ingredients, in-package, and much quicker than fermentation [8], making it a suitable structuring alternative for plant-based yogurts. However, there is little information on pressure-induced protein-based emulsion gels besides whey [9] and soy proteins [10], nor any data comparing them to dairy yogurts. Hence, using mung bean, chickpea, pea, lentil, and faba bean proteins as examples, this work compares the viscosity and viscoelastic properties of high pressure-structured plant protein gels, without and with sunflower oil to emulate skim and whole milk yogurts, to commercial dairy yogurts, and discusses the feasibility of using HPP to develop plant-based yogurts. 

## 2. Methods

### 2.1. Materials and Sample Preparation

Commercially available mung bean protein isolate (Gludodia^TM^, Fuji Oil Pte Ltd., Singapore), chickpea protein concentrate (Artesa^TM^, PLT Health Solutions, Morristown, NJ, USA), pea protein concentrate (Vitessence^TM^ Pulse CT 1552, Ingredion, Westchester, IL, USA), lentil protein concentrate (Vitessence^TM^ Pulse 2550, Ingredion, Westchester, IL, USA), and faba bean protein concentrate (Vitessence^TM^ Pulse CT 3602, Ingredion, Westchester, IL, USA) powders were kindly provided by the respective ingredient suppliers. Sunflower oil (Sunbeam, Sime Darby Plantation, Kuala Lumpur, Malaysia), plain skim (Chobani Pty Ltd., Dandenong South, VIC, Australia) and plain whole (Fage International S.A., Strassen, Luxembourg) milk Greek yogurts were obtained from a local supermarket. Greek yogurts were chosen as they contained higher protein content than regular yogurts. The composition of the protein powders, as provided by the manufacturers, is shown in the Supplementary Materials (Table S1). 

To mimic the skim and whole milk Greek yogurt compositions, the plant protein powders of mung bean (MB), chickpea (CP), pea (PP), lentil (LP), and faba bean (FB) were added to water, without and with sunflower oil (SO). A 12% (*w/w*) protein concentration was fixed for all formulations as it was around the minimum gelation concentration for the plant proteins and enabled comparison between samples [11,12,13,14], while the fat content was approximately 5% (*w/w*) for samples with sunflower oil. The composition information and labels for all ten formulations and the reference yogurts are shown in Table 1. 

To prepare the solutions, the plant protein powders were added to Milli-Q water and high-shear mixed at 20,000 rpm for 4 min (T25 digital UltraTurrax fitted with a S25N-18G dispersion tool, IKA Works Inc., Wilmington, NC, USA). Sunflower oil was added prior to mixing for the oil emulsion samples. The mixed solutions were filled in flexible storage bags and vacuum sealed. Each bag contained about 25 mL of sample. The packaged samples were stored overnight at 4 °C prior to HPP treatment. 

### 2.2. High Pressure Processing (HPP) 

The samples were high pressure-treated using a 300 L commercial HPP unit (Hiperbaric, Burgos, Spain). Samples were subjected to 600 MPa pressure for a 5 min hold time, which are typical food industry processing parameters and were also found to induce protein gelation [15,16]. The initial temperature of the pressurizing medium (filtered water) was 5 °C. The HPP-treated samples were then stored at 4 °C to minimize microbial activity and analyzed within 24 h. Two treatments were conducted for each formulation. 

### 2.3. Rheological Analyses

The viscosity and viscoelastic properties of the HPP-treated and reference yogurt samples were analyzed using an Anton Paar MCR 302 rheometer (Anton Paar Germany Gmbh) equipped with a temperature control system. A 50 mm diameter parallel plate configuration with an interplate gap of 1 mm was used. All experiments were conducted at 4 °C. Viscosity was measured using the steady state flow curve from 0.1 to 1000 s^−1^ with five points per decade. To compare between samples, viscosity values at a shear rate of 1 s^−1^ were reported. The viscoelastic properties (G’, G’’, and tan δ) of the samples were subsequently characterized with amplitude sweeps from 0.01 to 100% strain at a constant frequency of 1 Hz with eight points per decade. The storage modulus (G’) and loss factor (tan δ) at 0.1% strain was used to compare between samples within the linear viscoelastic region. Each of the two treatments was measured once, and all data are presented as the average of the two treatments. 

## 3. Results and Discussion

### 3.1. Viscoelastic Properties of the HPP-Treated Plant Protein Samples

**Figure 1:**
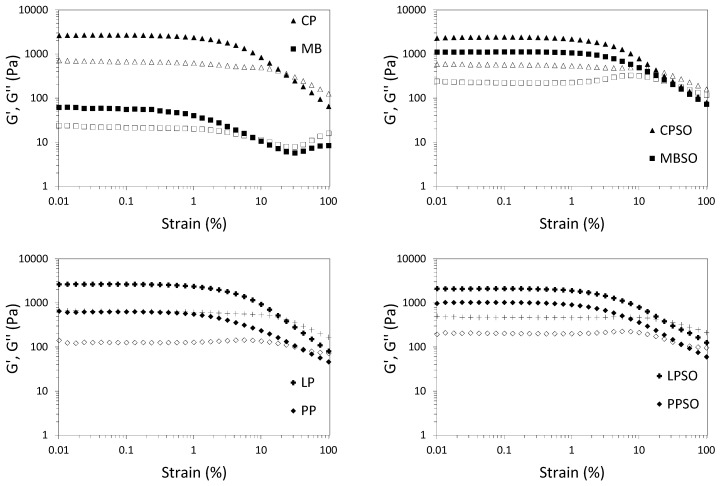

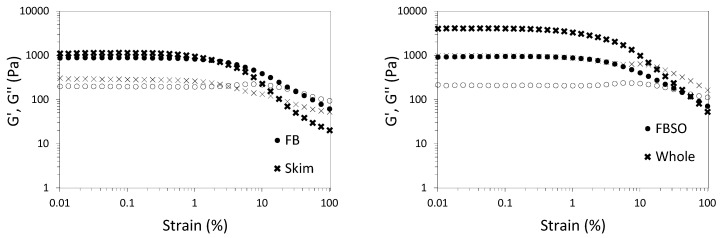
Amplitude sweeps of high pressure-treated 12% (*w*/*w*) mung bean (MB), chickpea (CP), pea (PP), lentil (LP) and faba bean (FB) protein samples without and with 5% (*w*/*w*) sunflower oil (SO) as compared to commercial plain skim and whole milk Greek yogurt. Storage modulus, G’ (closed symbol) and loss modulus, G’’ (open symbol). Each curve is the average of two treatments.

**Figure 2:**
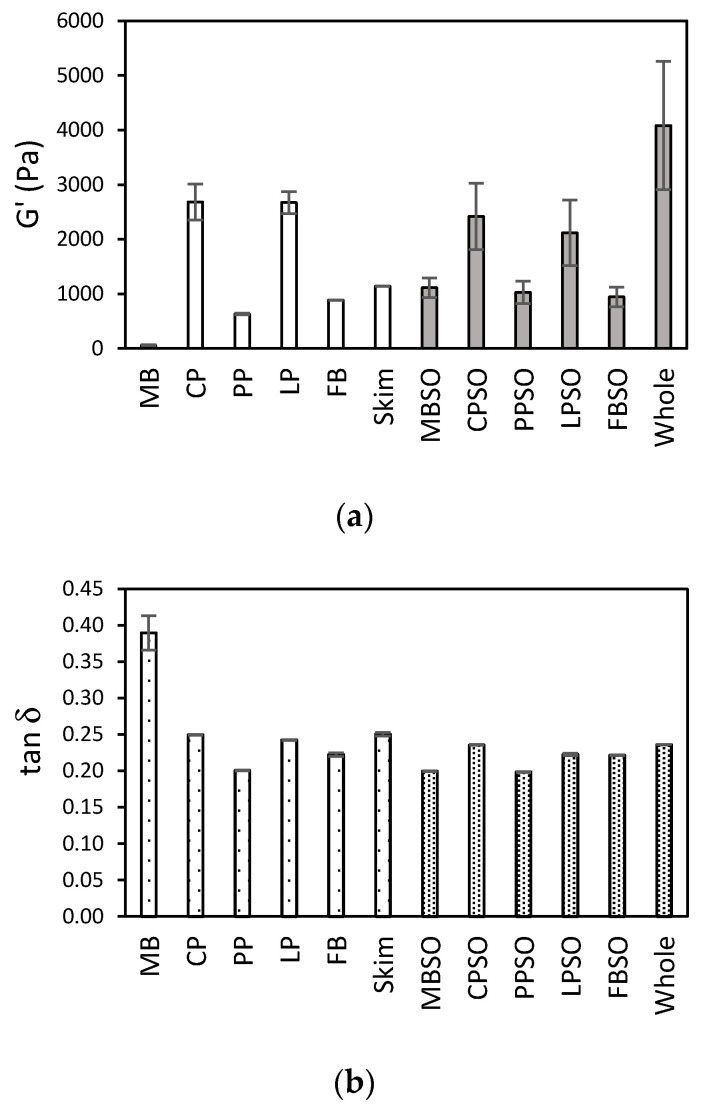

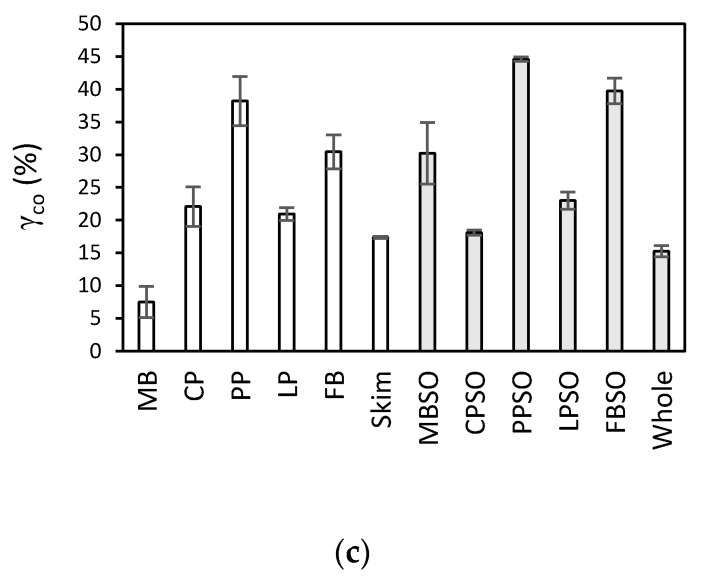
Rheological parameters obtained from amplitude sweeps of high pressure-treated 12% (*w/w*) mung bean (MB), chickpea (CP), pea (PP), lentil (LP), and faba bean (FB) protein samples without and with 5% (*w/w*) sunflower oil (SO) as compared to commercial plain skim and whole milk Greek yogurt. (**a**) Storage modulus (G’) at 0.1% strain; (**b**) loss tangent (tan δ) at 0.1% strain; (**c**) crossover strain (γ_co_) when G’ = G’’. The average of two treatments is presented with the error bar extremes showing the data for each treatment.

The amplitude sweep data are presented in Figure 1 and Figure 2. The values of G’ and G’’ indicate the solid-like and liquid-like character of the samples, respectively, while tan δ describes the ratio of viscous to elastic behavior. The breakdown of structure leading to flow can be represented by the crossover strain γ_co_ [17]. Overall, HPP formed viscoelastic gels (G’ > G’’) for all plant protein samples. The plant protein gels also had comparable gel strength (G’~10^2^–10^3^ Pa and tan δ~0.2–0.3) to commercial dairy yogurts except for MB (Figure 2a,b). However, the plant protein gels except for MB had moderately higher γ_co_ values than the dairy yogurts (Figure 2c), indicating their increased resistance to network breakdown. This could result in a texturally stable product without the need for added hydrocolloids, thereby contributing to a clean product label. 

There was variation in the viscoelastic properties between different plant proteins. The gel strength of the plant protein gels decreased in the order: CP~CPSO~LP~LPSO > MBSO~PPSO~FB~FBSO > PP >> MB (Figure 2a). While the variation could be attributed partly to different total solids content (Table 1), the type of plant protein could be more important. For example, although PP and LP had similar total solids content, LP and LPSO gels were considerably stronger than PP and PPSO, respectively. It was previously found that heat-treated lentil proteins generally formed stronger gels than pea proteins, but that depended on the pulse variety [18]. Hence, the choice of plant protein will be an important consideration to achieve the desired viscoelastic properties. In this study, FB and CPSO had the closest viscoelastic properties to the skim and whole milk yogurts, respectively, but more replicates are needed to establish statistical significance. Interestingly, the crossover strain values (Figure 2c) between plant proteins had an opposite pattern to gel strength (Figure 2a). This might indicate that the stronger protein network had a more brittle characteristic. Sensory evaluations are needed to determine if this brittle character can be detected. While not tested in this study, the blending of different plant proteins could enable the gel properties to be tailored for optimal sensory properties. 

The modest addition of sunflower oil led to little change in the viscoelastic properties for all plant protein samples except for MB and PP. The gel strength and crossover strain of MB and PP substantially increased when oil was incorporated in the MBSO and PPSO, respectively (Figure 2a,c). It was similarly reported that heat-induced pea protein gels were stiffer in the presence of oil [19]. With the addition of oil, it was likely that the protein molecules partitioned preferentially to the continuous phase, leading to a local increase in protein concentration. It was also established that the gel network is strengthened as protein-stabilized oil droplets act as fillers in the emulsion gel [20]. The difference in protein–protein and protein–lipid interactions might explain the variation in viscoelastic response between the plant proteins with the moderate addition of oil. While more in-depth study is needed to understand the role of lipids in HPP-formed emulsion gels, it should be noted that the oil content was fixed in this work to mimic full fat dairy yogurt. Furthermore, if similar viscoelastic properties and mouthfeel can be achieved without the addition of oil, that may lead to a lower caloric product.

### 3.2. Viscosity of the HPP-Treated Plant Protein Samples

**Figure 3:**
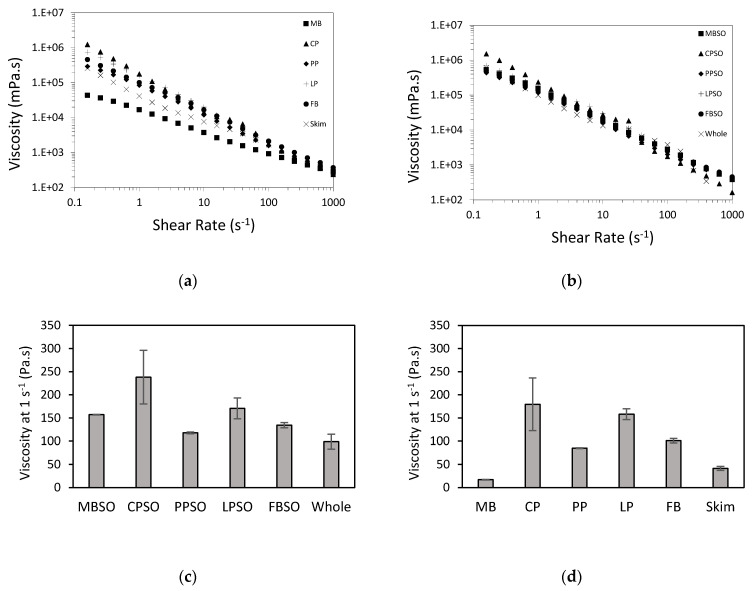
Flow curves (**a**,**b**) and viscosity at 1 s^−1^ (**c**,**d**) of high pressure-treated 12% (*w*/*w*) mung bean (MB), chickpea (CP), pea (PP), lentil (LP), and faba bean (FB) protein samples without and with 5% (*w*/*w*) sunflower oil (SO) as compared to commercial plain skim and whole milk Greek yogurt. Each curve is the average of two treatments. The average of two treatments is presented with the error bar extremes showing the data for each treatment.

The flow curve data are shown in Figure 3. All samples exhibited shear-dependent behavior likely due to the protein network breakdown and realignment of protein aggregates and oil droplets with increasing shear rate (Figure 3a,b). Except for the dairy yogurts, the plant protein samples viscosity (Figure 3c,d) mimicked the pattern of their gel strength (Figure 2a). The lower viscosity of the dairy yogurts could be due to its lower total solids content (Table 1). In this study, the emulsion gels also generally had higher viscosity than the hydrogels. Nonetheless, the viscosity values of the plant protein gels were in the same order of magnitude as the dairy yogurts. In this study, PP and PPSO had the closest viscosity to the skim and whole milk yogurts, respectively. 

### 3.3. Feasibility of Using HPP to Develop Plant-Based Yogurts

In this proof-of-concept study, the viscosity and viscoelastic properties of high pressure-structured plant protein gels have been shown to be comparable in behavior to commercial dairy yogurts, though further optimization and sensory evaluation is required. As different plant proteins exhibit variation in behavior, the gel properties could be simply tailored by blending different plant protein ingredients or by controlling pressure level [8]. Besides having customizable texture using minimal ingredients, the higher protein content and short processing time makes HPP an advantageous structuring method for plant-based yogurts.

**Figure 4:**
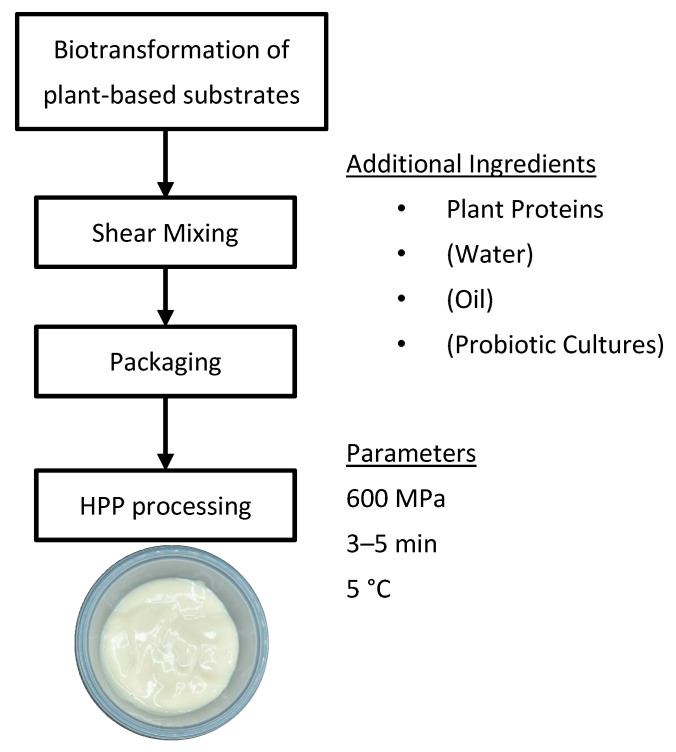
A proposed plant-based yogurt-making process using biotransformation for optimized flavor production and high pressure processing (HPP) for consistent texture generation. The bracketed ingredients are optional depending on formulation. The image shows HPP-structured mung bean yogurt (MBSO) in a cup.

The fermentation of plant-based substrates may still be needed for flavor production. Apart from having textural challenges in current plant-based yogurt-making, plant-based milks contain low levels of fermentable sugars and undergo inefficient fermentation [21,22]. Furthermore, the starter cultures may not match well to the highly variable plant-based milks from different sources [5,23]. Hence, by decoupling the flavor and texture generation of plant-based yogurts using separate biotransformation and HPP processes, a wider range of microorganisms could be explored to ferment the diverse sources of plant materials at lower cost and with higher yield and efficiency. This also allows for the valorization of low-value agricultural and waste materials such as plant fibers [24], husks [25], and peels [26,27], as fermentation substrates. Besides the characteristic dairy yogurt flavor volatiles like diacetyl and lactic acid [28] that can be produced and added, novel flavor compounds could also be explored. The subsequent HPP structuring step provides the consistency of texture regardless of plant-based flavor source. A potential plant-based yogurt-making process incorporating these ideas is shown in Figure 4.

An understandable concern is the viability of adding probiotic cultures before HPP as high pressures have been known to inactivate vegetative microorganisms [7]. A simple, but less economical solution, is to inoculate the plant-based yogurt with a higher probiotic culture concentration. Alternatively, since lower applied pressures (300–400 MPa) lead to partial protein denaturation and aggregation [8], such lower pressures might just be enough to create desired textures using strong structuring plant proteins such as CP and LP (Figure 2a), and also retain probiotic culture viability. In fact, it was found that high pressure treatment in the range of 200–300 MPa had minimal impact on *Bifidobacterium bifidum* and *Lactobacillus casei* probiotic strains in dairy yogurt [29]. However, attention needs to be paid to microbial safety as lower pressures also lead to reduced pathogenic microbial inactivation [7]. Another option is to use pressure-resistant probiotic cultures [30] and probiotic cultures in the form of pressure-resistant spores [31]. Finally, it is also possible to have no added cultures to create products equivalent to high protein pasteurized yogurts. 

## 4. Conclusions

In this proof-of-concept study, the viscosity and viscoelastic properties of high pressure-structured plant protein gels have been shown to be comparable in behavior to commercial dairy yogurts. To overcome the inefficiencies of current plant-based yogurt-making, a process involving separate biotransformation for optimized flavor production and high pressure processing for consistent texture generation was proposed. The next step is to test the proposal to effectively develop high protein plant-based yogurt products with desirable texture, flavor, and nutrition. 

## Figures and Tables

**Table 1:** The composition of 12% (*w*/*w*) protein solutions of mung bean (MB), chickpea (CP), pea (PP), lentil (LP) and faba bean (FB) without and with 5% (*w*/*w*) sunflower oil (SO), and reference plain skim and whole milk Greek yogurts from data provided by the manufacturers and tabulated using Microsoft Excel.

Formulation	% Protein (*w*/*w*)	% Fat (*w*/*w*)	% Carbohydrate (*w*/*w*)	% Sugars (*w*/*w*)	% Dietary Fiber (*w*/*w*)	% Starch (*w*/*w*)	% Ash (*w*/*w*)	% Moisture (*w*/*w*)
MB	12.0	<0.1	<0.9	n.a.	n.a.	n.a.	0.7	86.4
CP	12.0	0.2	6.1	n.a.	2.6	n.a.	0.7	81.0
PP	12.0	1.0	7.9	0.5	3.8	0.4	1.3	77.8
LP	12.0	1.0	7.3	0.4	3.1	0.9	1.2	78.5
FB	12.0	0.8	5.2	0.3	2.7	0.4	1.2	80.8
Skim	9.7	0.2	4.2	3.3	n.a.	n.a.	n.a.	<85.9
MBSO	12.0	5.0	<0.9	n.a.	n.a.	n.a.	0.7	81.4
CPSO	12.0	5.2	6.1	n.a.	2.6	n.a.	0.7	76.0
PPSO	12.0	5.0	7.9	0.5	3.8	0.4	1.3	73.8
LPSO	12.0	5.0	7.3	0.4	3.1	0.9	1.2	74.5
FBSO	12.0	5.0	5.2	0.3	2.7	0.4	1.2	76.6
Whole	9.0	5.0	3.0	3.0	n.a.	n.a.	n.a.	<83.0

The values with “<” symbol are estimated. n.a.: no available data.

## Supplementary Materials

The following are available online at https://www.mdpi.com/2304-8158/9/8/1126/s1, Table S1: The composition of mung bean (MB), chickpea (CP), pea (PP), lentil (LP) and faba bean (FB) protein powders from data provided by the manufacturers.
