# Supplementary material for: A Novel Approach to Structure Plant-Based Yogurts Using High Pressure Processing
Source: Foods. 2020 Aug 15;9(8):1126. doi: 10.3390/foods9081126 (PMC7466357; doi:10.3390/foods9081126)
Supplement: Supplementary file 1 [file foods-09-01126-s001.pdf]

Supplementary Data

Table S1. The composition of mung bean (MB), chickpea (CP), pea (PP), lentil (LP) and faba bean (FB) protein powders from data provided by the manufacturers.

**Protein powder**	**% protein (*w*/*w*)**	**% fat (*w*/*w*)**	**% carbohydrate (*w*/*w*)**	**% sugar (*w*/*w*)**	**% dietary fibre (*w*/*w*)**	**% starch (*w*/*w*)**	**% ash (*w*/*w*)**	**% moisture (*w*/*w*)**
MB	84.1	n.a.	n.a.	n.a.	n.a.	n.a.	4.7	5.0
CP	59.9	0.9	30.6	n.a.	13.0	n.a.	3.7	7.2
PP	51.7	4.3	34.1	2.1	16.5	1.9	5.7	4.2
LP	52.3	4.2	31.8	1.8	13.7	3.7	5.1	6.6
FB	59.5	3.7	25.9	1.7	13.4	1.9	5.7	5.2

n.a.: no available data.
